# Separation and Determination of Fe(III) and Fe(II) in Natural and Waste Waters Using Silica Gel Sequentially Modified with Polyhexamethylene Guanidine and Tiron

**DOI:** 10.1155/2017/8208146

**Published:** 2017-10-31

**Authors:** Svetlana Didukh, Vladimir Losev, Elena Borodina, Nikolay Maksimov, Anatoly Trofimchuk, Olga Zaporogets

**Affiliations:** ^1^Scientific Research Engineering Centre “Kristall”, Siberian Federal University, Krasnoyarsk, Russia; ^2^Institute of Chemistry and Chemical Technology, Siberian Branch, Russian Academy of Sciences, Krasnoyarsk, Russia; ^3^Taras Shevchenko National University of Kyiv, Kyiv, Ukraine

## Abstract

Silica gel, sequentially modified with polyhexamethylene guanidine and pyrocatechin-3,5-disulfonic acid (Tiron), was suggested for sorption separation and determination of Fe(III) and Fe(II). It was found that quantitative extraction of Fe(III) and its separation from Fe(II) were attained at pH 2.5–4.0, while quantitative extraction of Fe(II) was observed at pH 6.0–7.5. An intensive signal with *g* = 4.27, which is characteristic for Fe(III), appeared in EPR spectra of the sorbents after Fe(II) and Fe(III) sorption. During interaction between Fe(II) and Tiron, fixed on the sorbent surface, its oxidation up to Fe(III) occurred. Red-lilac complexes of the composition FeL_3_ were formed on the sorbent surface during sorption regardless of initial oxidation level of iron. Diffuse reflectance spectrum of surface complexes exhibited wide band with slightly expressed maxima at 480 and 510 nm. Procedures for separation and photometric determination of Fe(III) and Fe(II) at the joint presence and total Fe content determination as Fe(II) in waste and natural waters was developed. The limit of detection for iron was 0.05 *μ*g per 0.100 g of the sorbent. The calibration graph was linear up to 20.0 *μ*g of Fe per 0.100 g of the sorbent. The RSD in the determination of more than 0.2 *μ*g of Fe was less than 0.06.

## 1. Introduction

Element speciation, including determination of various oxidation states of the elements, in environmental objects is an important challenge of analytical chemistry. Iron refers to bioactive metals and plays an important biological role in plants, animals, and human beings. Natural waters contain iron in oxidation states +2 and +3, and Fe(III) content is much higher than Fe(II) content at that.

Photometric methods of analysis are the most widely used methods for determination of iron in various oxidation states [1, 2]; they are highly sensitive and selective. Organic reagents used in photometric methods form complex compounds with either Fe(III) or Fe(II). The best known organic reagents for the photometric determination of Fe(II) are N-heterocyclic bases: 1,10-phenantroline and 2,2′-dipyridyl [2]. Sulfosalicylic acid and Tiron [3] are the most widely used photometric reagents for Fe(III) determination.

Some reagents-derivatives of di-2-pyridyl ketone hydrazone: di-2-pyridyl ketone benzoylhydrazone and di-2-pyridyl ketone salicyloylhydrazone [4, 5] are known to form complex compounds with both Fe(II) and Fe(III). Detection of Fe(II) is carried out at one wavelength, and then the other wavelength is used for determination of the total iron content; Fe(III) content is calculated as a difference between total iron and Fe(II) content.

The following approaches are used for the photometric determination of Fe(II) and Fe(III) in one sample.

The first one is based on the application of the reagents which form complex compounds with Fe(II) or Fe(III). In this case, if the reagent forming complexes with Fe(II) is applied, the concentration of Fe(III) is calculated as a difference between total iron (after reduction of Fe(III) to Fe(II) using ascorbic acid, hydroxylamine, or reductor minicolumn) and Fe(II) content. If the reagent forming complexes with Fe(III) is applied, concentration of Fe(II) is calculated as a difference between total iron (after oxidation of Fe(II) to Fe(III) using hydrogen peroxide) and Fe(III) content [3, 6–8].

The second approach is based on the application of two chelating reagents, one of which is selective to Fe(II) and the other one is selective to Fe(III). For example, spectrophotometric sequential injection system was proposed for simultaneous determination of Fe(II) and Fe(III) based on introduction of reagents (1,10-phenanthroline and sulfosalicylic acid) into a stream of samples. The subsequent introduction of EDTA into a stream resulted in decomposition of Fe(III) compound with sulfosalicylic acid and absorption of Fe(II) compound with 1,10-phenanthroline was measured [9]. Fe(III) and Fe(II) were separated by silica microcolumn ion chromatography and determined via complexation with salicylic acid and 1,10-phenanthroline, respectively [10]. Capillary zone electrophoresis was applied for the simultaneous determination of iron(II) and iron(III) selectively complexed with 1,10-phenanthroline and trans-cyclohexane-1,2-diaminetetraacetic acid [11].

The third approach is based on different optical characteristics or different rates of formation of colored complexes of Fe(II) and Fe(III) with some organic reagents, for example, gallic acid [12] or Tiron [13].

Organic reagents are used in the combination of various methods of separation and determination of Fe(II) and Fe(III). Two-line manifold flow injection system with optoelectrochemical detection was used for separate determination of Fe(II) and Fe(III) [14]. Fe(III) was determined using photometric method as complex compound with sulfosalicylic acid, and Fe(II) was determined using electrochemical method. Method of separate determination of Fe(II) and Fe(III) using atomic absorption spectroscopy was suggested. Method is based on sorption separation of Fe(II) as its complex with ferrozine on a C_18_-modified silica column and direct atomic absorption determination of Fe(III) in solution passed through the column; then, Fe(II) was determined in eluate after desorption of iron(II)-ferrozine complex using atomic absorption spectroscopy [15].

Photometric method is used in coupling with sorption preconcentration in order to improve its sensitivity and selectivity. Iron may be determined directly in the sorbent phase [16–21] or in the solution after desorption [22–24]. Sorbents based on ion-exchange resins [16–18], polymethacrylate matrixes [20], silica [19, 24], cellulose [22], and naphthalene [23] are suggested.

Colorless sorbents are preferred to be used for sorption-photometric determination of Fe(III) and Fe(II). From this point of view silica based sorbents modified with colorless organic reagents which can form colored complexes with iron ions are very promising. Examples of such reagents include Tiron (4,5-dihydroxybenzene-1,3-disulfonic acid), which forms colored complex compounds with Fe(III) [25, 26].

Sorption of Fe(III) complexes with Tiron from aqueous solutions using ion-exchange resin AV-17 was studied in [27]. Fe(III) forms complex with Tiron in solution at pH of 3.5–9.0 and Fe(II) – at pH of 6.0–9.0; this phenomenon was used for sorption-photometric determination of Fe(III) at pH of 3.5, and the total content of Fe(II) and Fe(III) was determined at pH of 6–9.

At the present work silica gel sequentially modified with polyhexamethylene guanidine and Tiron was suggested for sorption separation and sorption-photometric determination of Fe(III) and Fe(II). Procedures for separate sorption-photometric determination of Fe(III) and Fe(II) from one sample of water and sorption-photometric and test-method for determination of the total iron content as Fe(II) in natural waters were developed.

## 2. Experimental

### 2.1. Reagents and Chemicals

All reagents were of analytical grade. Deionized water was used for the preparation of the solutions.

A stock standard solutions of Fe(III) and Fe(II) (100 mg L^−1^) were prepared by dissolving of FeSO_4_ and Fe_2_(SO_4_)_3_ in 0.1 M H_2_SO_4_. Working solutions with lower concentrations were prepared by dilution of stock solution with deionized water immediately prior to use.

The required pH was adjusted by adding HCl, NaOH, or acetic buffer solution (pH 4.0–6.5) and ammonium chloride buffer solution (pH 7.5–9.0). Hydroxylamine hydrochloride (0.1 M solution) was used in order to reduce Fe(III) into Fe(II).

Silica gel Silokhrom S-120 (fraction of 0.1–0.2 mm, specific surface area ~120 m^2 ^g^−1^, and average pore diameter ~45 nm) was used as a matrix for the sorbent synthesis.

Stock solution of polyhexamethylene guanidine hydrochloride (PHMG) (7.5% w/w solution) was prepared by dissolving weighted portion of BIOPAG-D reagent (Institute of Ecotechnological problems, Moscow, Russian Federation) in deionized water.

A 0.016 M Tiron stock solution was prepared by dissolving accurately weighted portion of the reagent in deionized water. Solutions with lower concentrations were prepared by dilution of the initial solution with deionized water.

### 2.2. Apparatus

Diffuse reflectance spectra (DRS) over the range of 380–720 nm were registered using Pulsar Spectrophotocolorimeter (Himavtomatika, Russia). Spectra were plotted against coordinates calculated using the Kubelka-Munk function; that is, *F*(*R*) = (1 − *R*)^2^/2*R* is wavelength (nm), where *R* is diffuse reflectance coefficient.

The UV-Vis spectra and absorbancy were registered using Cary 100 Spectrophotometer (Varian, Australia). Inductively coupled plasma optical emission spectrometer Optima 5300DV (Perkin-Elmer, USA) was used to determine metal ions concentration in solutions. The EPR spectra were recorded with an Elexsys E-580 instrument (Bruker, Germany). The pH measurements were carried out with a SevenEasy pH Meter S20 (Mettler-Toledo, Switzerland).

Peristaltic pump Masterflex L/S (Thermo Fisher Scientific, USA) was used for pumping solutions through a minicolumn with a sorbent.

### 2.3. Synthesis of SiO_2_-PHMG-Tiron Sorbent

SiO_2_-PHMG sorbent was synthesized according to procedure described in article [28]. Weighted portions of SiO_2_-PHMG (0.100 g) were placed into test-tubes with ground stoppers, 10 mL of Tiron solution of appropriate concentration was added, and the tube was stirred for 5 min. The resulting SiO_2_-PHMG-Tiron sorbent was separated from the solution by decantation and washed two times with deionized water. Tiron extraction was determined by the photometric analysis of water phase at the absorption band of the reagents *λ*_max_ = 292 nm (рН 1–7) and *λ*_max_ = 297 nm (рН > 8).

### 2.4. Preconcentration of Fe(II) and Fe(III) by SiO_2_-PHMG-Tiron

In the batch experiment Fe(II) or Fe(III) solution was placed into a graduated test tube with a ground stopper; 1.0 mL of 0.1 M hydroxylamine solution was added (in Fe(II) sorption experiment); NaOH, acetic (pH 4–6), or ammonium chloride (pH > 7) buffer solution was added to adjust required pH; and water was added to a total volume of 10.0 mL. SiO_2_-PHMG-Tiron sorbent mass of 0.100 g was added; the tube was stopped and stirred for 1–30 minutes. The solution was decantated, the sorbent moved into the fluoroplastic cell, and diffuse reflectance coefficient was measured. The distribution of iron was controlled by the analysis of water phase using inductively coupled plasma optical emission spectroscopy (ICP-OES).

**Figure 1:**
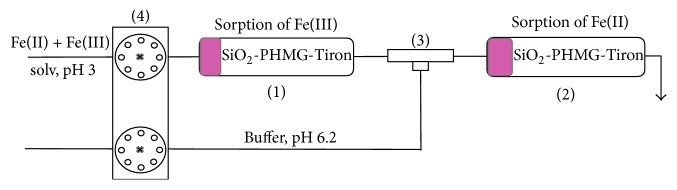
Scheme of the sorption separation of Fe(III) and Fe(II) in flow analysis using SiO_2_-PHMG-Tiron (minicolumn (1, 2), tee-joint (3), and peristaltic pump (4)).

A schematic diagram of the flow analysis system is shown in Figure 1. Two minicolumns (inner diameter 3 mm, height 50 mm) (1, 2) each filled with 0.100 g of SiO_2_-PHMG-Tiron sorbent were connected sequentially one after another via a tee-joint (3). Solution (20 mL) at pH 3 containing 1.0–5.0 *μ*g Fe(II) and 1.0–5.0 *μ*g Fe(III) in various ratios was pumped through the first minicolumn (1) at flow rate 0.5 mL min^−1^ using peristaltic pump (4). Acetic buffer solution with pH 6.2 was introduced continuously through the tee-joint (3). The resulting solution was pumped through the second minicolumn (2). Fe(III) was sorbed in the first minicolumn at pH 3.0, while Fe(II) was passed through the first minicolumn (1) and quantitatively extracted in the second one (2) at pH 6.2.

## 3. Results and Discussion

### 3.1. Tiron Fixation on the SiO_2_-PHMG Surface

**Figure 2:**
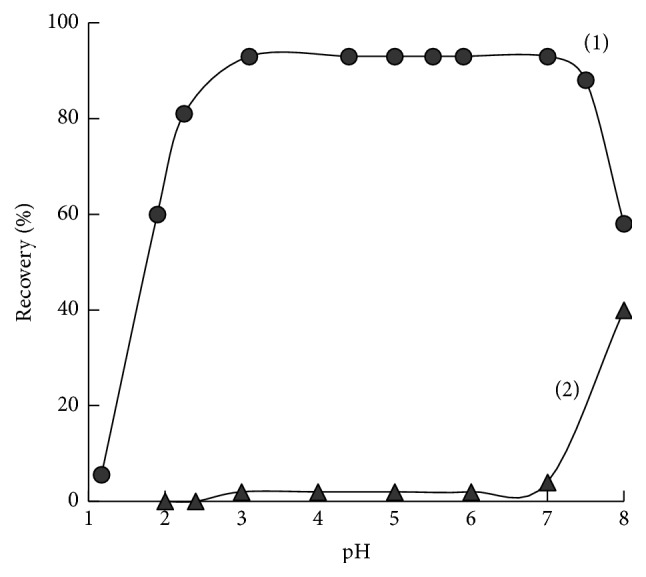
Recovery of Tiron (1) and catechol (2) by the SiO_2_-PHMG sorbent versus рН (*C*_Tiron_ = 0.16 mM (1, 2); 0.100 g of the sorbent; *V* = 10 mL, contact time 10 min).

Maximum recovery (≥98%) of Tiron from solution of 0.16 mM L^−1^ by SiO_2_-PHMG sorbent was attained at pH of 3.0–7.5 (Figure 2, curve (1)). The time of attainment of sorption equilibrium was less than 5 min. Tiron fixation on the surface of SiO_2_-PHMG occurs due to interaction between deprotonated sulfonic groups of the reagent and positively charged amine groups of PHMG. This assumption was confirmed by comparing the recovery curves of Tiron and its unsulfonated analog—catechol versus pH. In contrast to Tiron, catechol recovery by SiO_2_-PHMG sorbent (Figure 2, curve (2)) in the pH range of 2–7 did not exceed 1–3%.

**Scheme 1:**
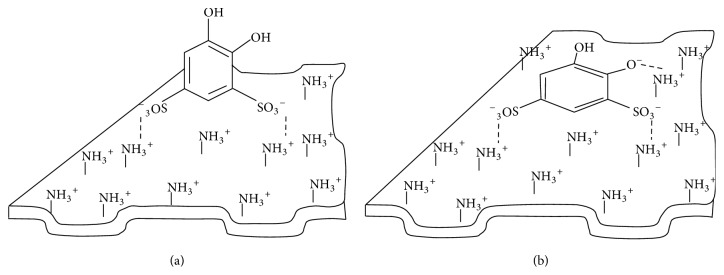
Expected arrangement of the Tiron molecules on the surface of the sorbent at рН 3 (a) and рН 6.0 (b).

**Figure 3:**
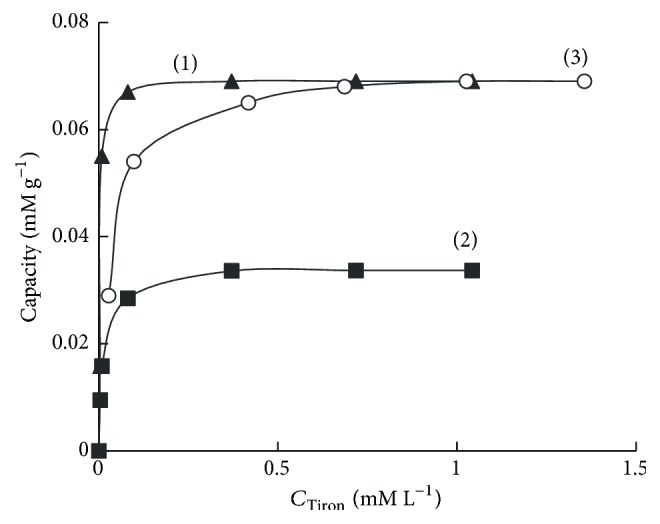
Sorption isotherms of SiO_2_-PHMG sorbent for Tiron at рН 3.0 (1), рН 6.0 (2), after sequential sorption of Tiron at рН 6.0, and then at рН 3.0 (3).

Maximum sorption capacity of SiO_2_-PHMG for Tiron was 69 *μ*mol g^−1^ at pH 3.0 and 33 *μ*mol g^−1^ at pH 6.0 (Figure 3, curves (1), (2)). The difference in sorption capacities connected with the fact that at pH 3 Tiron sorption proceeds due to electrostatic interaction between sulfonic groups of the reagent and amine groups of PHMG fixed on the silica surface; in this case Tiron molecule is arranged perpendicular to the sorbent surface (Scheme 1(a)). At pH 6.0 Tiron fixation occurs due to both electrostatic interaction of sulfonic groups of the reagent with amine groups of PHMG and interaction of deprotonated hydroxyl groups of the reagent (р*К*_*а*1_ = 7,7) with amine groups of PHMG, resulting in parallel arrangement of Tiron molecules to the surface of the sorbent (Scheme 1(b)).

This assumption is confirmed by the fact that during sequential treatment of SiO_2_-PHMG with Tiron solutions first at pH 6.0 and then at pH 3.0 an additional adsorption occurred, and total sorption capacity for Tiron was 69 *μ*M g^−1^ (Figure 3, curve (3)). This value coincides with the sorption capacity of the sorbent obtained at pH 3.0. When passing from pH 6.0 to pH 3.0 changes in Tiron arrangement proceed from parallel to perpendicular against the sorbent surface; this process leads to the release of seats (amine groups of PHMG) for additional fixation of Tiron molecules.

Treatment of SiO_2_-PHMG-Тiron sorbent obtained at pH 3.0 with solutions at pH 6.0 did not lead to the reagent desorption. This was confirmed by the absence of characteristic for Tiron absorption bands in solution.

Thus, for the sorbent with a maximum Tiron surface concentration it should be synthesized at pH 3.0.

Tiron fixation on the SiO_2_-PHMG surface is strong enough. Quantitative desorption of Tiron is achieved in 2 M HCl or in highly saline solutions (≥50 g L^−1^ of NaCl); this indirectly confirms electrostatic mechanism of fixation.

### 3.2. Fe(III) or Fe(II) Sorption by SiO_2_-PHMG-Tiron in the Batch Mode

**Figure 4:**
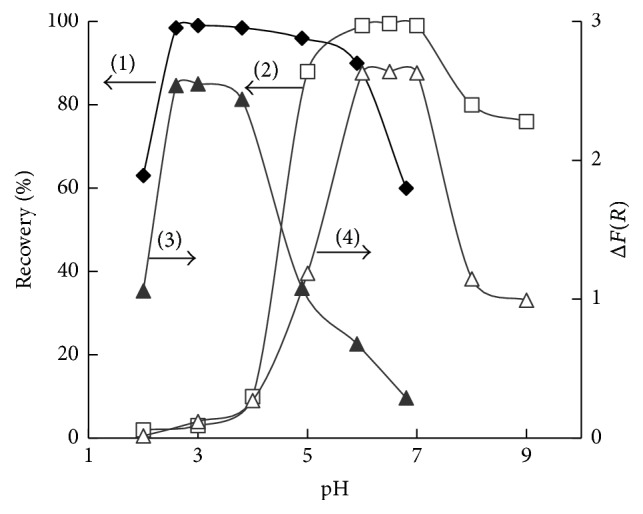
Extraction of metal Fe(III) (1) and Fe(II) (2) by SiO_2_-PHMG-Тiron sorbent and Δ*F*(*R*) of the sorbent after sorption of Fe(III) (3) and Fe(II) (4) versus pH (0.100 g of the sorbent; *C*_Tiron_ = 16 *μ*mol g^−1^; *C*_Fe_, *μ*g mL^−1^: 1,0 (1, 2), 0,5 (3, 4); *V* = 10 mL).

Maximum Fe(III) recovery (98-99%) by SiO_2_-PHMG-Tiron sorbent was observed at pH of 2.5–4.0 and that of Fe(II) at pH of 6.0–7.5 (Figure 4, curves (1), (2)). Decrease in the recovery of Fe(III) at pH > 4 was connected with its hydrolysis. Decrease in the recovery of Fe(II) at pH < 6 is coincided with conditions of its interaction with Tiron in aqueous solution. Recovery of Fe(II) at pH 3.0 was less than 1-2%. The time of attainment of sorption equilibrium of Fe(III) (at pH 2.5–4.0) and Fe(II) (at pH 6.0–7.5) extraction did not exceed 10 min.

**Figure 5:**
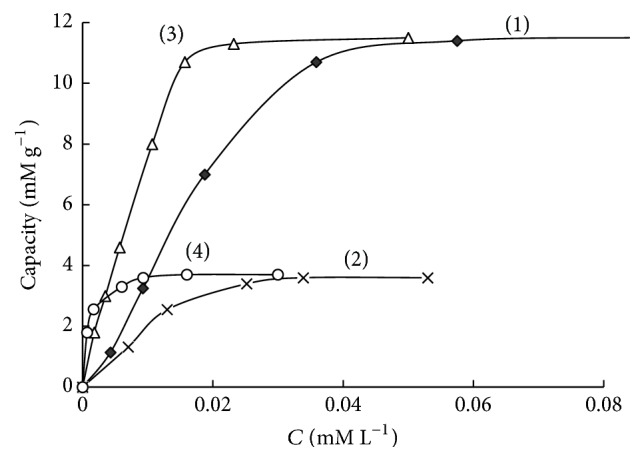
Sorption isotherms of SiO_2_-PHMG-Тiron sorbent for Fe(III) (1, 2) and Fe(II) (3, 4) (рН: 3.0 (1, 2), 6.2 (3, 4); 0.1 М NH_2_OH (2, 4), *C*_Tiron_ = 33 (1, 3), 9.2 (2, 4) *μ*М g^−1^).

Sorption capacity for Fe(III) determined from the horizontal section of the sorption isotherms of SiO_2_-PHMG-Tiron sorbent with the surface concentration of Tiron 33 *μ*mol g^−1^ and 9.2 *μ*mol g^−1^ was 12 *μ*mol g^−1^ and 3.6 *μ*mol g^−1^, respectively (Figure 5, curves (1), (2)). Similar values of sorption capacity of SiO_2_-PHMG-Tiron sorbent were obtained for Fe(II) (Figure 5, curves (3), (4)). The data indicate that during Fe(III) and Fe(II) sorption complexes with the ratio Fe : Tiron ~ 1 : 3 are mainly formed on the surface of the sorbents with different surface concentration of Tiron.

**Figure 6:**
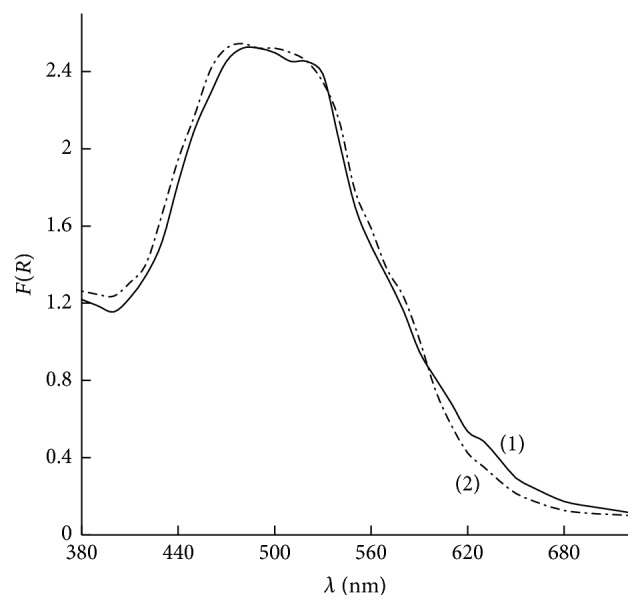
Diffuse reflectance spectra of the surface complexes after Fe(III) (1) and Fe(II) (2) sorption by SiO_2_-PHMG-Tiron sorbent (рН: 3.0 (1), 6.2 (2), *C*_Fe_ = 0.5 *μ*g mL^−1^; *V* = 10 mL, 0.100 g of the sorbent).

During Fe(III) sorption at pH 2.5–4.0 the sorbent surface acquired a red-lilac color. The DRS was a wide band with slightly expressed maxima at 480 and 510 nm (Figure 6, spectrum (1)). It is known that in aqueous solutions Fe(III) forms complexes with Tiron with the stoichiometry 1 : 1, 1 : 2, or 1 : 3 [25, 26]. Blue complex FeL (*λ*_max_ = 665 nm) is formed at pH < 3.5, violet complex FeL_2_ (*λ*_max_ = 553 nm) is formed in the pH range of 3.5–6.5, and red-lilac complex FeL_3_ (*λ*_max_ = 480 nm) is formed at рН ≥ 6.5.

After comparison of the maxima in the DRS of Fe(III) surface complexes with the maxima of their absorption spectra in aqueous solutions it could be assumed that complexes of Fe(III) with Tiron with the composition FeL_3_ are mainly formed on the surface of the SiO_2_-PHMG-Tiron sorbent. FeL_3_ complex is formed at the pH values that are characteristic for FeL and FeL_2_ complexes formation in solutions because SiO_2_-PHMG surface promotes an additional coordination of FeL and FeL_2_ surface complexes with Tiron molecules.

Similar shift of FeL_3_ complex formation in acid area with expended pH range of its formation up to 4–8 was observed during interaction of Fe(III) with Tiron on the surface of anion-exchange resin Amberlyst A-27 [29].

During Fe(II) sorption in the pH range of 6.0–7.5 at both presence and absence of 0.001–0.1 М hydroxylamine solution the surface of SiO_2_-PHMG-Tiron sorbent acquired a red-lilac color. DRS of the sorbent after Fe(II) sorption from solutions with pH 6.0–7.5 was identical to DRS of the sorbent after Fe(III) sorption at pH 2.5–4.0 and had slightly expressed maxima at 480 and 510 nm (Figure 6, spectrum (2)).

Intensities of the bands in DRS of the sorbents after sorption of Fe(III) and Fe(II) were equal, which is evidence of identity of the surface complexes composition.

Maximum intensity of the sorbent color was observed in the pH range coinciding with the pH ranges of the quantitative extraction of Fe(III) and Fe(II) (Figure 4, curves (3), (4)).

### 3.3. Study of Fe(III) Complexes with Tiron on the Surface of SiO_2_-PHNG-Tiron Sorbent and in Aqueous Solutions Using EPR

In order to determine the oxidation state of iron in its complexes with Tiron using EPR method at 77 K the following objects were studied:

- SiO_2_-PHMG-Tiron sorbents after Fe(III) and Fe(II) sorption at various pH values;
- solutions after mixing of Fe(III) and Fe(II) solutions with Tiron at various pH values.

**Figure 7:**
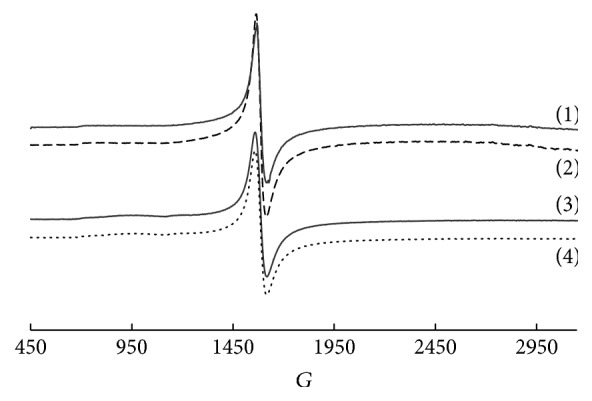
EPR spectra of Fe(III) complexes formed on the surface of SiO_2_-PHMG-Тiron sorbent during the sorption of Fe(III) (1) and Fe(II) (2) and Fe(III) complexes with Tiron in aqueous solution after interaction between Fe(III) (3) or Fe(II) (4) and Tiron (рН: 3.0 (1), 6.2 (2), and 8.0 (3, 4), 0.200 g of the sorbent (1, 2),and *C*_Fe_ = 20 *μ*g per 0.200 g of the sorbent (1, 2), 0.36 mМ (3, 4); *C*_Tiron_ = 16 *μ*mol g^−1^ (1, 2); 1.6 mМ (3, 4)).

In the low-field region of EPR spectra of SiO_2_-PHMG-Tiron sorbent after Fe(III) and Fe(II) sorption an intensive signal with *g* = 4.27 was observed (Figure 7, curves (1), (2)). Similar EPR signal was observed for Fe(III) complexes with desferrioxamine [30]. EPR spectra of SiO_2_-PHMG-Tiron after Fe(III) and Fe(II) sorption in optimum conditions were identical, which is an evidence of the oxidation state of iron within the surface complex +3. On the basis of EPR data it can be concluded that during interaction of Fe(II) with Tiron at pH 6.0–7.5 on the surface of SiO_2_-PHMG-Tiron it is oxidized up to Fe(III).

Even though Fe(III) complexes with Tiron of different composition (Fe : Tiron = 1 : 1, 1 : 2, 1 : 3) are formed in solution at different pH values, EPR spectra of solutions after mixing Fe(III) and Tiron solutions (Figure 7, curve (3)) in the pH range of 3.0–9.0 are identical to each other and to EPR spectra of Fe(III) complexes, which are formed on the surface of SiO_2_-PHMG-Tiron sorbent and characterized by intensive EPR signal with *g* = 4.27. EPR spectra of the solutions after mixing Fe(II) and Tiron solutions at pH 6.0–9.0 are also characterized by intensive signal with *g* = 4.27 (Figure 7, curve (4)). The shape of the spectra and EPR signals intensities are identical for the solutions obtained by mixing of the same concentrations of Fe(III) or Fe(II) with Tiron. Even in the presence of 0.001–0.1 M hydroxylamine in the solution Fe(II) formed complex with Tiron at pH 6.0–7.5 having intensive signal with *g* = 4.27 in EPR spectrum. The identity of ESR spectra in this case indicates that hydroxylamine does not prevent oxidation of iron (II) during complexation with Tiron at pH 6.0–7.5.

Thus, from the EPR data it can be concluded that during interaction of Fe(II) with Tiron both in solution and on the surface of the sorbent it is oxidized to Fe(III). Both dissolved in water oxygen and the reagent itself can be an oxidant of Fe(II) [13]. The rate of Fe(II) oxidation increases with the rising of degree of saturation of solution with oxygen at pH > 5 in the presence of acetate ions; that coincides with the area of its quantitative extraction by SiO_2_-PHMG-Tiron.

The identity of EPR spectra of SiO_2_-PHMG-Tiron sorbent after Fe(III) and Fe(II) sorption is the evidence of oxidation level of iron within the surface complex +3; and the identity of color and DRS of Fe(III) complexes on the surface of SiO_2_-PHMG-Tiron sorbent is the evidence of identical composition of the surface complexes.

### 3.4. Sorption-Photometric Determination of Fe(III) and Fe(II) Using SiO_2_-PHMG-Tiron

As the iron content on the sorbent surface increased, the intensity of sorbent color increased proportionally; and shape of DRS and position of its maxima did not depend on iron concentration. Formation of intensively colored complexes on SiO_2_-PHMG-Tiron surface was used for development of the following procedures:

- Sorption-photometric determination of Fe(III) and Fe(II)
- Sorption-photometric determination of total iron in natural waters.

The analytical characteristics of the developed method such as the limit of detection, linear range, and correlation coefficient were obtained by processing standard solutions under optimum conditions. A linear calibration graph was obtained for the determination of iron (II) under the proposed experimental conditions. The calibration equations in coordinates Δ*F*(*R*) − *c*, where *c* is iron content (*μ*g per 0.100 g of the sorbent), were as follows:

- Δ*F*(*R*) = (0.593 ± 0.003)*c* (*R*^2^ = 0.999) for Fe(II) sorption.
- Δ*F*(*R*) = (0.596 ± 0.003)*c* (*R*^2^ = 0.998) for Fe(III) sorption.

The detection limit for iron determination calculated using 3s-criterion was 0.05 *μ*g per 0.100 g of the sorbent. The calibration graphs were linear up to 20.0 *μ*g of Fe per 0.100 g of the sorbent. The relative standard deviation (RSD = ($s/\overset{-}{x}$) × 100%) in the determination of more than 0.2 *μ*g of Fe per 0.100 g of the sorbent was less than 6.2%, *n* = 5 (Table 1). The detection limits, the range of linearity of calibration graphs, and RSD are independent of initial oxidation level of iron.

SiO_2_-PHMG-Tiron sorbent is characterized by good kinetics. As the ratio volume of solution to the sorbent mass (*V* : *m*) rises from 10^2^ to 10^3^, and the time of attainment of sorption equilibrium did not exceed 10 min. An increase of volume of solution from 10 to 100 mL (using 0.100 g of the sorbent) leads to decrease of the relative detection limit from 5 ng mL^−1^ to 0.5 ng mL^−1^.

### 3.5. Effect of Potentially Interfering Ions

Solutions containing Fe(II) or Fe(III) (0.1 *μ*g mL^−1^) and other ions were prepared and the developed procedure was applied in order to determine the selectivity of the sorbent.

Sorption preconcentration from solution at pH 3.0 and sorption-photometric determination of Fe(III) was not affected by the following cations (in multiple amounts): Na^+^, K^+^, Sr^2+^, Сa^2+^, Мg^2+^ (1000), Рb^2+^, Ni^2+^, Zn^2+^, Hg^2+^ (500), Bi^3+^, (500), Sn^2+^, Al^3+^, Cr^3+^, and Cu^2+^ (100).

Sorption preconcentration from solution at pH 6.2 and sorption-photometric determination of Fe(II) was not affected by the following cations (in multiple amounts): Na^+^, K^+^, Sr^2+^, Сa^2+^, Мg^2+^, Рb^2+^ (1000), Ni^2+^ (500), Zn^2+^ (250), Bi^3+^, Hg^2+^ (100), Sn^2+^, Al^3+^ (50), Cr^3+^, and Cu^2+^ (10). Salt background up to 50 g L^−1^ for NaCl, 5 g L^−1^ for Na_2_SO_4_, and 25 g L^−1^ for Na_2_SO_3_ did not prevent Fe(III) and Fe(II) preconcentration and determination.

Selectivity of Fe(III) determination is higher compared to Fe(II) determination because Fe(III) complexation with Tiron occurs in more acidic area, where no interaction with other metal ions (forming complexes at pH > 4) with Tiron takes place [31, 32].

### 3.6. Sorption Separation and Determination of Fe(III) and Fe(II)

Dependence of Fe(II) and Fe(III) quantitative extraction by SiO_2_-PHMG-Tiron sorbent versus pH and formation of intensively colored Fe(III) surface complexes was used for sequential sorption isolation and separate determination of Fe(III) and Fe(II) from one sample of the solution.

During sorption in the batch mode Fe(III) content was found almost 1.5 times higher than it was added and Fe(II) content 1.5 times lower than it was added. But the total Fe(III) and Fe(II) content were equal to when they were added. Overestimated results of Fe(III) determination and underestimated results of Fe(II) determination are explained by saturation of solution by atmospheric oxygen during intensive stirring at pH 3, the optimum conditions for Fe(III) extraction, and Fe(II) is oxidized up to Fe(III).

Sorption in flow analysis using minicolumn allows eliminating saturation of solution by atmospheric oxygen and accomplishing both separation and determination of Fe(II) and Fe(III) from one sample of solution by the length of colored zone of the sorbent using system represented on Figure 1. The sorbent in minicolumns became red-lilac color.

The length of colored zone of the sorbents after passing Fe(III) and Fe(II) solutions of equal concentrations was equal, and it increased proportionally to their content in solution. The calibration function for Fe(II) and Fe(III) determination by the length of the colored zone (*l*) was as follows: *l*  (mm) = 2*c* ± 1, where *c* is iron content in minicolumn, *μ*g. Iron content determined by the length of the colored zone in the model solution is represented in Table 2. An increase of the flow rate of the solution from 0.5 to 3.0 mL min^−1^ led to the erosion of the colored zone.

Procedure for the separate determination of Fe(III) and Fe(II) by the length of the colored zone in minicolumn was used for the analysis of well water during storage: in 30 min, 6 h, and 24 h after sampling. Obtained results are represented in Table 3.

In well waters that are of high iron content (>2 mg L^−1^) and free of organic compounds (humic and fulvic acids) in contact with air oxidation of Fe(II) to Fe(III) proceeds with subsequent precipitation of slightly soluble iron (III) hydroxide. Fe(II) content was determined in well waters with high iron content after separation of iron (III) hydroxide sediment using membrane filter.

The data represented in Table 3 shows that in the cases of low iron content in natural waters the results of sorption-photometric determination of total content of Fe(III) and Fe(II) are in agreement with the results of total iron determination using ICP-OES.

### 3.7. Determination of Total Iron in Natural Waters

In order to decompose metal complexes with organic compounds natural waters were boiled with nitric acid. Organic complexes of iron were destroyed, and iron was oxidized up to Fe(III) as a result, which was hydrolyzed forming poorly soluble compounds. Despite higher selectivity of sorption-photometric determination of Fe(III) at pH 3.0, in order to determine the total iron content it is reasonable to reduce Fe(III) to Fe(II), because it is less hydrolyzed in aqueous solutions and does not form poorly soluble compounds. Hydroxylamine is preferred to be used as the reducing agent because its presence does not affect the formation of the surface complex of Fe(III) with Tiron.

Developed procedure was applied for total iron determination in waste waters (samples number 1 and number 2), taken in various districts of Krasnoyarsk city; river water; drink waters: low mineralized water “Uchumskaya” and highly mineralized water “Zagorie” produced in Krasnoyarsk Krai. The accuracy of procedure was confirmed by ICP-OES method. The results of iron determination are represented in Table 4.

An intensely colored zone appeared when a sample was passed through a minicolumn filled with a sorbent. Dependence of the length of colored zone of the sorbent on iron content was used for total iron determination in natural waters (Table 4).

## 4. Conclusion

SiO_2_-PHMG-Tiron sorbent proposed for preconcentration, separation, and determination of Fe(II) and Fe(III) is characterized by simplicity of synthesis from widespread and available reagents and does not require complex and expensive equipment. The sorbent allows quantitative sequential isolation and determination of Fe(II) and Fe(III) from one sample of water. Developed procedures are comparable to FAAS and ICP-OES in terms of detection limits. Application of minicolumn filled with SiO_2_-PHMG-Tiron sorbent allows rapid and accurate visual estimation of Fe(II) and Fe(III) content in natural waters. Procedure of iron determination by the length of colored zone in minicolumn does not require equipment and can be applied for iron determination in natural waters in the field.

## Figures and Tables

**Table 1:** RSD for determination of iron concentration per 0.1 g of SiO_2_-PHMG-Tiron (*n *= 5).

Added, *μ*g	Found, *μ*g	RSD, %
0.10	0.09 ± 0.01	9.1
0.20	0.21 ± 0.02	6.2
0.50	0.50 ± 0.02	4.0
1.0	1.00 ± 0.04	3.2
5.0	5.1 ± 0.3	4.5

**Table 2:** Results of Fe(III) and Fe(II) determination in model solutions using minicolumn (*n* = 5).

Added, *μ*g		Length of colored zone, mm		Found, *μ*g	
Fe(III)	Fe(II)	Fe(III)	Fe(II)	Fe(III)	Fe(II)
—	5.0	—	10 ± 1	—	5.0 ± 0.5
5.0	—	10 ± 1	—	5.0 ± 0.5	—
1.0	5.0	2 ± 1	10 ± 1	1.0 ± 0.5	5.0 ± 0.5
2.0	5.0	4 ± 1	10 ± 1	2.0 ± 0.5	5.0 ± 0.5
5.0	1.0	10 ± 1	2 ± 1	5.0 ± 0.5	1.0 ± 0.5
5.0	2.0	10 ± 1	4 ± 1	5.0 ± 0.5	2.0 ± 0.5
5.0	5.0	10 ± 1	10 ± 1	5.0 ± 0.5	5.0 ± 0.5

**Table 3:** Results of Fe(III) and Fe(II) determination in well waters (*n* = 5).

Sample	Found, *μ*g mL^−1^						Total Fe, *μ*g mL^−1^
	Fe(III)			Fe(II)			
	0.5 h	6 h	24 h	0.5 h	6 h	24 h	
Well water number 1	0	0.14	*∗*	0.18	0.05	0	0.18
Well water number 2	0.1	1.0	*∗*	1.7	0.6	0.1	1.8

^*∗*^Iron (III) hydroxide precipitation.

**Table 4:** Results of total iron determination in natural and mineral waters (*n* = 5).

Sample	Found Fe, mg L^−1^		
	Sorption-photometric method	Column method	ICP-AES
Waste water No. 1	1.8 ± 0.1^a^	2.0 ± 0.5^a^	1.6 ± 0.1
Waste water No. 2	2.1 ± 0.1^a^	2.0 ± 0.5^a^	2.1 ± 0.1
River water	0.60 ± 0.04^a^	0.5 ± 0.5^a^	0.60 ± 0.03
Mineral water «Uchumskaya»	0.045 ± 0.008^a^	~0.05^a^	0.050 ± 0.007
Mineral water «Zagorie»	14.4 ± 0.7^b^	—	15.0 ± 0.6

^a^Sample volume: 5 mL. ^b^Sample volume: 1 mL.
